# Skin Barriers in Dermal Drug Delivery: Which Barriers Have to Be Overcome and How Can We Measure Them?

**DOI:** 10.3390/pharmaceutics12070684

**Published:** 2020-07-20

**Authors:** Christian Gorzelanny, Christian Mess, Stefan W. Schneider, Volker Huck, Johanna M. Brandner

**Affiliations:** Department of Dermatology and Venerology, Center for Internal Medicine, University Medical Center Hamburg-Eppendorf, 20246 Hamburg, Germany; c.gorzelanny@uke.de (C.G.); c.mess@uke.de (C.M.); st.schneider@uke.de (S.W.S.); v.huck@uke.de (V.H.)

**Keywords:** skin barrier, drug delivery, stratum corneum, tight junctions, claudin, microscopy, spectroscopy, tomography, TEER, TEWL, atopic dermatitis

## Abstract

Although, drugs are required in the various skin compartments such as viable epidermis, dermis, or hair follicles, to efficiently treat skin diseases, drug delivery into and across the skin is still challenging. An improved understanding of skin barrier physiology is mandatory to optimize drug penetration and permeation. The various barriers of the skin have to be known in detail, which means methods are needed to measure their functionality and outside-in or inside-out passage of molecules through the various barriers. In this review, we summarize our current knowledge about mechanical barriers, i.e., stratum corneum and tight junctions, in interfollicular epidermis, hair follicles and glands. Furthermore, we discuss the barrier properties of the basement membrane and dermal blood vessels. Barrier alterations found in skin of patients with atopic dermatitis are described. Finally, we critically compare the up-to-date applicability of several physical, biochemical and microscopic methods such as transepidermal water loss, impedance spectroscopy, Raman spectroscopy, immunohistochemical stainings, optical coherence microscopy and multiphoton microscopy to distinctly address the different barriers and to measure permeation through these barriers in vitro and in vivo.

## 1. Introduction

Optimization of drug delivery to the exact compartment and biophase where the drug is needed is an important goal to increase effectivity and to decrease side effects. To achieve this, it has to be known which mechanical barriers the drug and its delivery system have to cross. In the skin there are the mechanical barriers of the stratum corneum (SC) [1] and the tight junctions (TJs) in the interfollicular epidermis [2,3], and in hair follicles (HFs) [4]. Furthermore, there is putatively a mechanical barrier at the basement membrane at the dermal–epidermal junction and barriers in glands and blood vessels. In addition, barrier function in particular skin conditions should be known as mechanical barriers are often affected in the course of skin diseases such as atopic dermatitis. Therefore, advanced methods to denote these barriers and to determine their exact localization as well as their tightness or leakiness to (marker) molecules or ions of different sizes and charges are necessary. Ideally, the methods can also measure concentrations of the (marker) molecules/ions overcoming the barrier. The markers can be taken as surrogate molecules for topically applied drugs. Optimally, the drugs themselves (or their delivery system) can be detected by the method. This is especially true for the investigation of molecules on the way from skin surface into the skin (outside-in). However, also the passage of tracer molecules from inside-out and the relevant barriers on their path are of interest to understand the various barriers of the skin in more detail. In addition, because the SC, which is the first barrier to molecules from outside-to inside, is often the rate-limiting barrier for uptake of topically applied molecules (see below) and thus prevents these molecules from reaching further barriers in the viable epidermis and below, it can only be seen by inside-out barrier function assays to what extent the other skin structures provide additional mechanical barriers. This is important to understand the complex skin barrier system in general and to comprehend what limits the loss of water and solutes from the body. Furthermore, the additional barriers may be of importance when the SC is impaired, e.g., in certain skin diseases, or for molecules which are not primarily stopped in the SC.

The methods to investigate skin barriers and their relevance for outside-in and inside-out passage of molecules can be divided into (electro) physical, chemical and microscopic methods. Importantly, it is not only necessary to know the potential of a method but also to be aware of its (current) limitations. Is a method e.g., able to measure the passage through a specific barrier or does it ‘only’ measure the flow through the whole epidermis/skin? Can it be applied merely in vitro/ex vivo, which means in (3D) cell cultures or excised skin or also in vivo?

In this review, we give an overview about the various mechanical barriers in the skin and describe methods used to measure barrier function/molecular flow such as transepidermal water loss (TEWL) measurements, Raman spectroscopy and multiphoton microscopy. As an example for a skin disease with altered barrier function, we describe changes in mechanical barriers in atopic dermatitis (AD).

The knowledge of the composition and structure of the skin barriers and their barrier function to surrogate markers is important for the development of new drug delivery systems, especially also when planning to deliver the drugs to certain compartments of the skin and for drug delivery in diseased skin. Of course, for this planning and optimization also detailed knowledge of the drug and its delivery system itself is important. Physicochemical properties such as diffusion and partition properties, as well as pharmacokinetics and pharmacodynamics independent from skin barrier interactions are crucial. In addition, the right choice of the biological and mathematical experimental model, the proper administration and sampling of the drug as well as sample preparation/detection techniques to quantify the drug are of high relevance. However, these topics are beyond the scope of this review. For a good overview of many of these aspects see [5]. Moreover, we will focus here on mechanical barriers of the skin, even though the microbiome barrier and the immunological barrier also play an important role in drug delivery, especially also concerning side effects.

## 2. Which Skin Barriers Have to Be Overcome?

### 2.1. Interfollicular Epidermis

#### 2.1.1. Stratum Corneum (SC)

**Figure 1:**
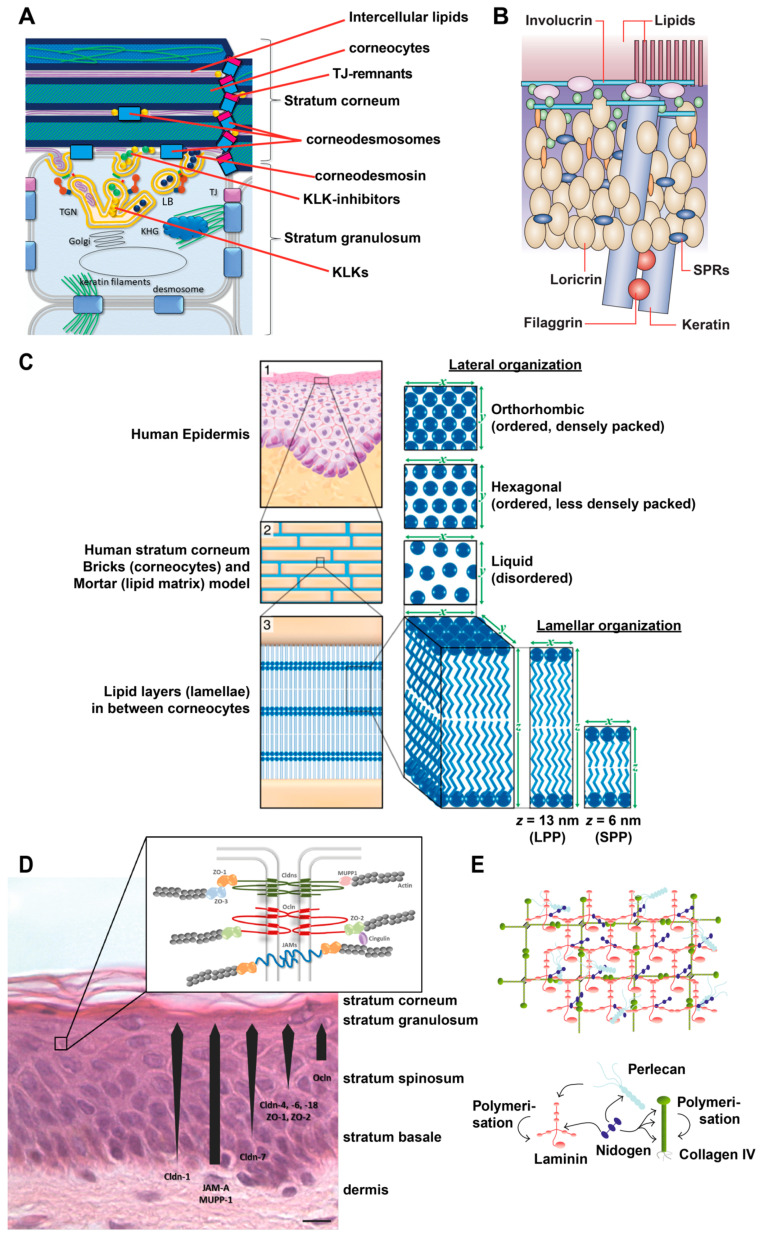
Overview of epidermal molecular structures important for skin barrier function. (**A**) Schematic drawing of the uppermost *stratum granulosum* and *stratum corneum*. KHG: keratohyalin granula KLK: kallikrein-like kinases, LB: lamellar body, TGN: trans-golgi-net TJ: tight junctions. (**B**) Schematic drawing of the cornified envelope. SPR: small proline-rich. (**C**) Organization of the intercellular lipids of the *stratum corneum*. LPP: long periodicity phase, SPP: short periodicity phase. (**D**) Tight Junction (TJ) structure and TJ proteins in the epidermis. Cldn: claudin, JAM: junctional adhesion molecule, Ocln: Occludin, ZO: zonola occludens protein. (**E**) Composition and structure of the basement membrane, (**A**) modified from [19], (**B**) from [20], (**C**) from [21], (**D**) from [2], (**E**) from [22].

The SC is the first mechanical barrier bordering the environment. It is highly relevant for the absorption process of the vast majority of drugs by passive diffusion. It consists of corneocytes which are connected via corneodesmosomes and TJ remnants, and intercellular lipids [1,6] (see Figure 1A).

Corneocytes are terminally differentiated keratinocytes. They are characterized by a lack of cell nuclei and organelles, an accumulation of cytokeratin filaments which are bundled, among others, by filaggrin, and by the presence of a rigid cornified envelope (CE). The CE is built by several proteins such as involucrin, loricrin, small proline-rich proteins (SPRs), envoplakin, periplakin, filaggrin and cysteine protease inhibitor A (cystatin A), which are cross linked by transglutaminases [1,6,7] (see Figure 1B).

Corneodesmosomes are composed, among others, by desmoplakin, desmoglein 1, desmocollin 1 and corneodesmosin. Corneodesmosin is delivered to the extracellular space by lamellar bodies at the granular cell layer and is then integrated into desmosomes [8] which are transformed step by step to corneodesmosomes (see Figure 1A). Proper degradation of corneodesmosomes is essential for the desquamation of corneocytes and thus for an ordered turnover of the epidermis. The degradation of corneodesmosomes is performed by proteases such as Kallikrein-related peptidases (KLKs) and cathepsins (see Figure 1A). They are controlled by protease inhibitors, e.g., lymphoepithelial-Kazal-type 5 inhibitor (LEKTI), cholesterol sulfate and pH [1]. In addition, it is thought that TJ remnants (see below) restrict the access of proteases to corneodesmosomes [6,9] (see Figure 1A). This might explain why corneodesmosomes at the lateral sides of the cells, where TJ remnants are present, are degraded later than corneodesmosomes at the basal and apical sides.

The extracellular area of the SC is filled with densely packed lipid layers, so called lipid lamellae. They consist of cholesterol, free fatty acids and ceramides. They form two crystalline lamellar phases, the long periodicity phase (LPP) with a repeat distance of ca. 13 nm and the short periodicity phase (SPP) with a repeat distance of about 6 nm [10,11]. Furthermore, also the lateral packing is of importance. In healthy skin, most SC lipids are present in a dense orthorhombic packing, while a subpopulation adopts a less dense hexagonal packing. In addition, a liquid-crystalline packing exists which allows the lipids the greatest freedom of movement [12,13,14] (see Figure 1C). The precise 3D organization depends on the composition of lipids, especially ceramides, which in turn defines barrier function [14,15,16]. Furthermore, skin hydration and temperature (as well as solvents and penetration enhancers, see below) influence the lateral packing of the SC lipids [17].

The lipids are mainly derived as precursors delivered by lamellar bodies (see Figure 1A). Yet, also sebaceous glands and extracutaneous sources have been described to contribute lipids to the SC [16,18]. Additionally, the lipid processing enzymes are mainly secreted via lamellar bodies.

The importance of the SC as a whole for skin barrier function has been shown for decades in many reports. First of all, removal of several layers of the SC, e.g., by tape stripping, results in impaired skin barrier function, shown by e.g., increased TEWL and enhanced uptake of externally applied substances [23,24]. Thus, removal of the SC by tape stripping or laser abrasion is also a method to enhance drug delivery [25,26,27]. In addition, several further methods to open the SC have been applied to improve transdermal drug delivery, such as thermal ablation, electroporation, sonophoresis, iontophoresis, fractional laser ablation, microneedles, and high velocity jets [25,27,28]. However, many of these methods do not only open the SC but also involve deeper layers of the epidermis or hair follicles.

Various distinct components of the SC such as filaggrin, CE-proteins and corneodesmosin have been shown to be involved in skin barrier function.

Filaggrin mutations have been related to defective barrier function in patients with ichthyosis vulgaris and AD [29,30]. Filaggrin-deficient mice exhibit increased desquamation under mechanical stress and increased antigen penetration [31]. The absence of filaggrin impairs corneocyte surface texture and stiffness [32]. However, basal TEWL is not elevated in filaggrin knock-out mice [31] and epidermal equivalents of filaggrin-null keratinocytes do not show impaired outside- in and inside-out skin barrier function [33]. Therefore, absence of filaggrin appears to only predispose the skin for barrier impairment whereas further damages (e.g., mechanical stress) are required to induce a relevant barrier disruption. It also has to be taken into account that filaggrin is not only involved in structural organization of the corneocytes but its degradation products urocanic acid and pyrrolidine carboxylic acid contribute to an acidic skin pH and retention of water [34,35], which are important for proper barrier formation (see below).

Several proteins involved in CE formation have been shown to be associated with skin diseases characterized by impairment of skin barrier function. Mutations or down-regulation of SPR, SPR3 and loricrin have been linked to AD [36,37,38]. Mutations in loricrin are associated with loricrin keratoderma [38] loss-of-function mutation in SCTA (cystatin A) can induce autosomal recessive exfoliative ichthyosis and acral peeling skin syndrome [39,40]. Studies in mice show that knock-down of single components of the CE such as envoplakin, periplacin, involucrin and loricin often have no or only a very mild impact on CE structure and skin phenotype, hinting for strong redundancy and thus for the importance of the CE itself [41,42]. Consequently, loss of the crosslinking enzymes transglutaminase 1 and 5 which affect several CE proteins results in perturbation of CE’s and barrier function in mice [43] and have been associated with Acral peeling skin syndrome (missense mutation in TGM5) and lamellar ichthyosis (mutation in TGM1 resulting in deficiency) [44,45,46].

Mutations in genes coding for the corneodesmosomal proteins corneodesmosin and desmoglein 1 result in skin barrier diseases such as generalized inflammatory types of peeling skin syndrome and severe dermatitis, multiple allergies, and metabolic wasting (SAM) syndrome [6,47]. A knock-out of corneodesmosin results in impairment of epidermal barrier function and is lethal in neonatal mice [48].

The importance of lipids for skin barrier function can be seen by the fact that removal of lipids from the SC, e.g., by acetone, increases TEWL [49]. In addition, it was shown that abnormal lipid organization due to changes in lipid composition result in impaired epidermal barrier function [15]. Changes in free fatty acids and cholesterol seem to have only a minor influence on the barrier function, whereas ceramides have been described to be most relevant [1]. For example, changes in ceramide levels, composition and chain lengths were found in AD and were associated with barrier impairment [50,51]. Many drug delivery enhancers address the lipid compartment of the SC. For instance, chemical penetration enhancers such as fatty acids (e.g., oleic acid and linoleic acid) and surfactants such as sodium lauryl sulphate are used [27,52,53,54]. Drug delivery formulation can perturb the SC lipids and therefore SC barrier e.g., by having high solvent concentrations which remove skin lipids. Furthermore, components of the formulation can intercalate into the structured lipids and therefore decrease diffusional resistance. In addition, they can modify the solubility parameter of the skin lipids [55].

Besides these structural components of the SC, also SC hydration has to be considered for maintenance of skin barrier function. In addition to SC lipid organization and the size of corneocytes, natural moisturizing factor (NMF) is a major determinant of SC hydration [15]. NMF is derived by the degradation of filaggrin (see above) but also sweat contains NMF [56]. SC hydration is important for skin plasticity and SC morphology [57]. Furthermore, it influences the activities of various proteases involved in desquamation and lipid synthesis. Thus, alteration of skin hydration during short- and long-term drug delivery may influence SC barrier function.

Finally, also skin pH affects several factors regulating epidermal barrier integrity, such as proteases important for desquamation and enzymes important for lipid synthesis [35,58]. But also in deeper layers, microenvironmental pH is important for proper folding of molecules and therefore optimal interaction, e.g., in TJs [59]. Thus, next to its direct physicochemical effect on the applied drug or drug carrier system itself, changes in pH during drug delivery may be exploited to change skin barrier function but may also cause side effects.

#### 2.1.2. Tight Junctions

TJs form a continuous barrier in the stratum granulosum (SG) of the epidermis. They are the second barrier to molecules on their paracellular passage from outside to inside.

TJs in the epidermis form a barrier to molecules of different sizes with the smallest tested molecules being Biotin-SH with 556 Da. Dependent on their composition, TJs and especially claudins can block the passage of compounds in a charge-selective way. Therefore, they reduce the paracellular passage of ions such as chloride, sodium and calcium [60,61].

TJs consist of three families of transmembrane proteins: claudins, TJ associated MARVEL-proteins (including occludin and tricellulin) and junctional adhesion molecules. Especially claudins are important for defining the barrier function of the TJs. In human epidermis, predominantly the presence of claudin-1 and claudin-4 has been demonstrated (see Figure 1D). These are barrier tightening claudins [62]. In addition, TJs contain a variety of plaque proteins, such as ZO-1, -2, cingulin, and atypical proteinkinase C, which are important for scaffolding, regulation and signaling and establish TJs as a signaling platform [63] (see Figure 1D).

The importance of TJs to skin barrier was impressively shown by the death of claudin-1 knock-out mice at the first day of birth due to excessive water loss [64]. More detailed investigations have shown that the absence of claudin-1 results in a leakage of the TJs present in the SG for small molecular tracers up to 5000 Da but not for larger molecules (approx. 30 kDa) [64,65]. Interestingly, increased loss of water was due to an impaired SC and not primarily due to increased TJ water permeability [60,66]. Nonetheless, this also indicated that dysregulation of TJs affects the formation of the SC [67].

Complete loss of claudin-1 in human results in the neonatal ichthyosis sclerosing cholangitis (NISCH) syndrome. The disease is characterized by vulgar type ichthyosis, hypotrichosis with alopecia and sparse eyelashes/eyebrows with varying extents. In some patients, changes in the SC were shown [68,69,70,71,72]. To our knowledge, skin barrier function tests addressing this rare disease have not been published yet.

In AD lesional skin there is a strong downregulation of claudin-1 [73,74,75] and, depending on the cohort investigated, no, mild or medium downregulation in non-lesional skin [74,75,76,77]. Concerning barrier function, there is strong impairment of TJ barrier in lesional and no impairment in AD non-lesional skin with no or only mild downregulation of claudin-1 [74], reflecting the dose dependency of TJ barrier function on claudin-1 levels [74,78]. There is a slight upregulation of occludin and claudin-4 in non-lesional AD skin [74,75] while there is an upregulation of occludin and an altered localization of claudin-4 in lesional skin [74].

Several approaches to enhance drug delivery via modification of TJ proteins, especially claudin-1, have been described. Application of a TJ-disrupting peptide patch addressing claudin-1 results in barrier disruption as measured by increased TEWL [79]. When using theses peptides in combination with an epicutaneous influenza vaccination patch, immune response was increased.

The C-terminal part of *Clostridium perfringens* enterotoxin (cCPE) removes claudin-3, -4, -6 and -9 from TJs and was shown to be effective in impairing TJ ion [80] and molecular tracer [67,80] barriers in reconstructed human epidermis. In addition, it weakened also the SC barrier [67].

m19, a TJ binding peptide addressing claudin-1, -2, -4, and -5 reduces the transepithelial electrical resistance (TEER) (increases ion permeability) in normal human epidermal keratinocytes (NHEKs). 7A5, a monoclonal antibody directed to claudin-1 reduces TEER and increases 4 kDa Dextran flux. 3B11, a monoclonal antibody directed to claudin-4 also increases ion permeability in NHEKs [81].

The AT1002 peptide consists of six amino acids (FCIGRL) and can open TJs of the granular cell layer by leading to phosphorylation of the TJ structural protein ZO-1. AT1002 enhances the delivery of topically applied siRNA in mice, and its efficacy at treating skin diseases such as AD by delivering relevant siRNAs was tested in AD mouse models [82,83].

A broader approach by using sodium caprate, which opens TJs but also changes the SC results in decrease of TEER of reconstructed human epidermis [84].

These examples show that addressing TJs is a promising approach to enhance drug delivery. But it also indicates that unintended alterations of TJs by drugs or their carrier systems might be relevant.

#### 2.1.3. Basement Membrane (Basal Lamina)

The basement membrane (BM) is localized at the basal side of the *stratum basale* at the dermo–epidermal junction. It is an assembly of different matrix proteins and carbohydrates. Major components are e.g., laminins and collagens, proteoglycans such as perlecan, and hyaluronic acid. They form, together with a variety of further molecules, a cross-linked mat-like structure [22,85] (see Figure 1E) which is important for proper formation of the epidermis and consequently also for barrier formation. Auto-antibodies directed to laminin result in blistering pemphigoid diseases [86,87]. In atopic dermatitis, it was shown that thickness of the BM is significantly reduced [88].

The extent to which the BM can be considered as a barrier is largely unknown. The mesh-structure of the BM suggests that the exchange of substances between the epidermis and the dermis is attenuated. However, in inside-out barrier experiments, the intended transport of proteins till 40 kDa (HRP) was apparently not affected [89]. Nonetheless, the epidermal uptake of particles with a size of about 8 nm was significantly decreased [90,91]. Furthermore, the passage of virus particles, e.g., herpes simplex virus was stopped by the BM [92]. In addition, it has been reported that due to its strong negative charge the BM acts as a charge-selective barrier for larger (approx. 450 kDa) molecules [93].

### 2.2. Hair Follicles

Hair follicles (HFs) are complex structures present all over the human body except for glabrous skin. HFs undergo cycles with a consecutive sequence of anagen (growth phase), catagen (regression phase) and telogen/exogen (resting phase) [94]. The majority of HFs are in anagen, while fewer are in catagen and telogen. Even though flux in the HF is predominantly from inside-out, uptake of substances via HFs was shown and drug delivery by the route of HFs is of considerable interest [4,95,96].

**Figure 2:**
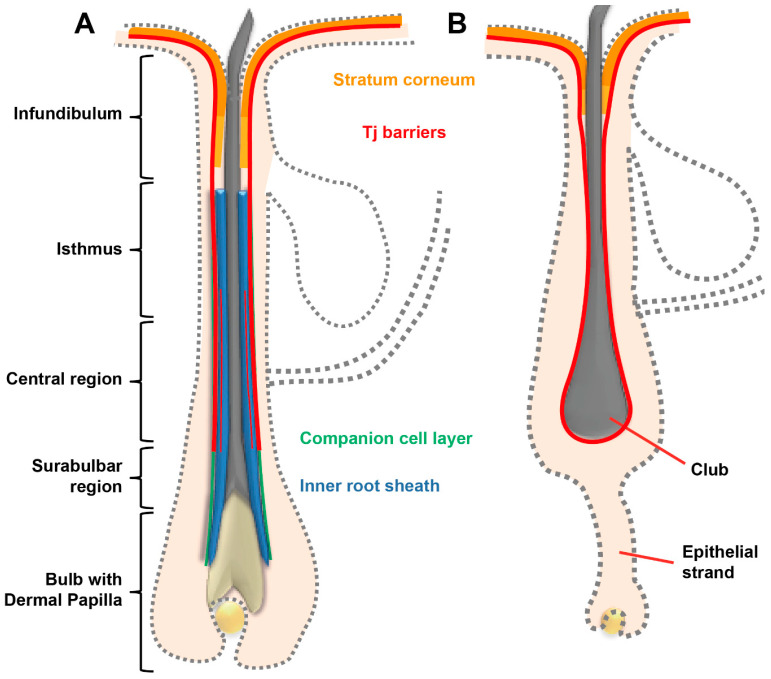
Schematic drawing of a hair follicle in anagen (**A**) and catagen (**B**) phase denoting stratum corneum (SC) and tight junction (TJ) barriers. More intense color of SC denotes SC similar to epidermal SC. Less intense color denotes infundibular SC with slightly different properties. Please note that the companion cell layer in the central and isthmus region is overlayed by the TJ barrier labelling.

Human anagen HFs contain two main barriers. Barrier-forming TJs are continuously present from the infundibulum down to the lower central part of the outer root sheath of the HF [97] (see Figure 2A). In the infundibulum, these TJ-containing layers are covered by a SC which is continuous to the SC of the epidermis. However, composition is slightly different [98]. In addition, there exist barrier-forming TJs between Henle and Huxley’s layers [97] (see Figure 2A). Concerning drug delivery, especially the upper part of the HF is accessible for drugs. Therefore, SC and TJs in this area may be predominantly relevant for drug uptake. Whether TJs between Henle and Huxley’s layer are of significance for outside-in barrier is a challenging question for future investigations with sophisticated microscopical tools.

TJ proteins/mRNAs demonstrated in human and porcine HFs up to now are ZO-1, ZO-2, occludin, claudin-1, and claudin-4 with also minor mRNA expression of claudin-12 and claudin-17 [97,99,100,101]. The distribution of TJ proteins depends on the specific areas of the HF from distal to proximal (infundibulum, isthmus region, central region, suprabulbar region and bulb) as well as from outside (bordering the environment) to inside (inner root sheath, outer root sheath) [97]. Barrier-forming TJs are found in layers with a colocalization of all investigated TJ proteins, e.g., in the SG of the infundibulum and in the companion cell layer of the outer root sheath of isthmus, central region and upper suprabulbar region [97]. TJ structures were confirmed in the companion cell layer in ultrastructural experiments [102].

In AD, Cldn-1 expression is decreased in HFs of lesional skin. In addition, down-regulation of Cldn-1 in keratinocytes derived from HFs results in decreased barrier function to ions as well as to 4 kDa Dextrans [97]. NISCH syndrome, a rare human genetic disease due to complete absence of claudin-1, is often accompanied by alopecia and reduced eyebrows/lashes (see Section 2.1.2). However, this is likely not only caused by changes in HF barrier function since Cldn-1 also regulates proliferation and apoptosis in HF keratinocytes [97].

In catagen and telogen HFs, TJs are found in the outermost living layer bordering the environment. Here, they are present all around the club hair [97] (see Figure 2B). In addition, in the infundibulum a SC is present [97].

Drug delivery into and via hair follicles seems especially promising for drugs loaded into nanocarriers [4].

### 2.3. Glands

In general, also skin glands form barriers. Because of the inside-out flux direction of dermal glands these routes are not preferably addressed for drug delivery, however, uptake into glands can be derived via certain techniques, e.g., iontophoresis.

Due to the presence of TJs (see below) in glands, improved transepidermal drug delivery by e.g., TJ barrier modulating enhancers may also attenuate the barriers in glands. Accordingly, this might result in side effects such as increased or decreased sweat flow or disturbed secretion of lipids via sebaceous glands.

#### 2.3.1. Sebaceous Glands

Sebaceous glands are multicellular exocrine glands that end in the hair shaft. They were shown to contain TJ proteins claudin-1, claudin-4, claudin-7, tricellulin and ZO-1. The sebaceous gland TJs form a barrier to intradermal injected anti-desmoglein-1 single chain variable fragment and lanthanum in mouse skin [103]. Holocrine secretion occurs outside of the TJ barrier. Knock-out of claudin-1 causes leakage of the TJ barrier in sebaceous glands and incomplete degradation of the plasma membrane and nuclei during holocrine secretion [103].

#### 2.3.2. Sweat Glands

Additionally, sweat glands contain a TJ barrier. On the ultrastructural level, the presence of barrier-forming TJs in sweat glands of human skin has already been shown in the 1970s and 1980s [104,105,106]. Sweat glands express occludin, claudin-1, claudin-3, claudin-4, claudin-10b and claudin-15 as well as ZO-1 and ZO-2 in human skin with varying expression patterns depending on the localization within the gland [107,108,109]. Claudin-1 is often absent from the barrier-forming TJs, while claudin-3, claudin-10 and claudin-15 seem to be more prominent. Of note, in mouse skin, only claudin-3, claudin-4 and claudin-10 are present [109].

Claudin-3 knock-out mice show TJ leakage and—putatively due to decreased retention of the sweat in the lumen of the upper sweat gland—less sweat [109]. Humans with less claudin-10b in sweat glands due to a missense mutation in the CLDN10B gene, exhibit anhidrosis. Related experiments in 3D cultures modelling sweat secretion showed that an impaired TJ strand formation perturbs paracellular sodium transport [108]. Patients with AD show decreased sweat volume and decreased expression of claudin-1, claudin-3, and claudin-15 [109].

Jajack et al. [110] showed that flux of biomarkers into sweat (e.g., glucose) can be increased by more than 10 times by using citrate, a calcium chelator which opens TJs, in combination with reverse iontophoresis to drive the negatively charged chelator down into the lumen of the sweat gland [110].

### 2.4. Blood Vessels

The ‘last barrier’ of the skin is its vasculature. Ending up in the papillary loops of the superficial arteriovenous plexus nearby to the dermo–epidermal junction in the upper dermis, a one-cell thick endothelial cell layer represents the interface between the surrounding tissue of the skin and the human vascular system. The role of the endothelium in the skin is analogue to its role in the whole body: it actively responds to pressure, shear forces, osmolarity, heat, chemokines and cytokines by modulation of its permeability and induction of also vegetatively controlled vasodilatation or constriction [111].

Next to the direct impact of e.g., inflammatory stimuli on the permeability, the skin vasculature exhibits a physiologically relevant noteworthiness: it constitutes the major effector component of thermoregulation by opening up vascular loops usually closed under resting conditions. Thus, the total skin ranges from a perfusion of 0.05 L/min upon cold stress via 0.25 mL/min in the mean under normo-thermic resting conditions to more than 5.00 L/min upon hyperthermia [112]. In addition to the thermoregulatory function, this meaningful modification of local perfusion rates affects the flux rate of substances outside-in and inside-out and therefore the barrier function of the skin [113,114]. For transdermal drug delivery, a plethora of clinical trials have already been successfully performed focusing on the heat induced increase of systemic plasma concentrations of topically applied substances such as fentanyl [115], clonidine [116], testosterone [117] or nicotine [118].

Summarized, contiguous to the epidermal components of the skin barrier, the blood vessel system of the skin has to be taken into consideration for an appraisal of the entire skin barrier function.

## 3. And How Can We Measure Them?

As outlined above, skin barrier depends on a large variety of distinct structures such as lipids, structural proteins and protein assemblies including corneodesmosomes and TJs. The barrier of the skin is therefore largely dependent on a plethora of different factors with different physical and chemical properties. Prerequisite for optimized delivery of drugs or carrier systems is therefore detailed knowledge on the molecular and structure-molecular composition of the skin barriers. In addition, monitoring of the flux of the drug/carrier system in relation to the various barriers is desirable. Although high-resolution microscopy (e.g., electron microscopy) offers the maximal information on barrier structures and localization of certain tracers, its application is currently limited to the analysis of certain time points in thin tissue sections sliced from skin biopsies without being able to measure dynamic changes. The same is true for approaches addressing single barrier-forming structures combined with tracer assays in light microscopy (e.g., TJ protein staining combined with biotinylation assays) which can only be performed ex vivo/in vitro in processed tissues. In contrast, low resolution imaging (e.g., optical coherence tomography, ultrasound), spectroscopic approaches (e.g., Raman spectroscopy) or transepidermal water loss (TEWL) measurements allow the investigation of the native skin barrier as a whole in vivo and at different time points whereas the contributions of distinct barrier-forming components such as SC or TJs cannot specifically be analyzed. Recent developments in the field of multiphoton microscopy offer high-resolution imaging in vivo which allows the longitudinal analysis of the skin with a micrometer resolution. Moreover, multiphoton tomography could be combined with fluorescence life-time imaging or Coherent anti-Stokes Raman spectroscopy combining high resolution imaging with powerful spectroscopy. Similarly, progress in optical coherence tomography and optoacoustic imaging envisions sophisticated tools that enable morphological examination of the skin and the tracking of drugs at high resolution.

In the following section, we briefly present the use of common methods to investigate skin barrier function. We will highlight their usability to address the different barriers of the skin for outside-in and for inside-out permeation. In addition, the possibility of flux-quantification and the in vivo applicability will be summarized (see also Table 1).

### 3.1. Physical/Chemical Methods

**Figure 3:**
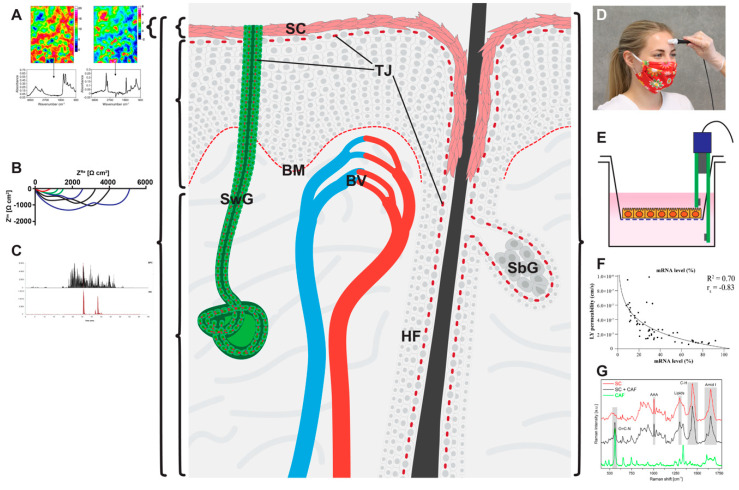
Localization of mechanical barriers in the skin (light red: stratum corneum (SC), Tight junctions (TJs) and basement membrane (BM)) and graphical representations of the various physical/chemical methods described in this review: (**A**) Fourier transform infrared (FTIR) spectroscopy (**B**) electrical impedance spectroscopy, (**C**) chemical analyses, (**D**) transepidermal water loss (TEWL), (**E**) transepithelial electrical resistances (TEER), (**F**) dye permeation analyses, and (**G**) Raman spectroscopy. Brackets denote the area measured by a specific method not discriminating between the different barriers within this area. (**B**) and (**C**) can be used to measure complete skin but can also be used—by additional preparation steps or more sophisticated methods—to discriminate between SC, viable epidermis and dermis. BV: blood vessel, HF: hair follicle, SbG: sebaceous gland, SwG: sweat gland. (**A**) from [122], (**C**) from [123], (**F**) from [74], (**G**) from [124].

The various methods described in this chapter are summarized in Table 1. Figure 3 gives an overview of the areas of skin comprised by the measurements with the various physical/chemical methods and the distinct barriers localized in these areas.

#### 3.1.1. Transepidermal Water Loss (TEWL)

The measurement of the TEWL is a well-established method to determine the amount of water that permeates through the skin, which means through interfollicular epidermis, HFs and glands. The recent review by Alexander et al. [119] summarizes the principles of TEWL measurements. TEWL measurements were frequently applied to quantify disease-related skin dehydration occurring e.g., in atopic skin. In this context, increased TEWL is mechanistically linked to an impaired skin barrier. For instance, the molecular correlation between skin barrier impairment and TEWL has been shown in the skin of AD patients with mutated filaggrin [120]. Other reports suggest a correlation between the skin lipid composition and the regulation of the TEWL [15]. Recently, TEWL measurements were used to quantify the opening of the skin barrier by penetration enhancers and the clear correlation between increased TEWL and enhanced accumulation of the model drug cidofovir was shown [121]. However, TEWL is of course not a direct measurement of the drug.

This method is very valuable to measure bulk changes of the skin barrier for inside-out movement of water as a surrogate for other molecules over time. It can easily be used in vivo when environmental influences are controlled.

Yet, TEWL measurements cannot be connected to single molecular structures but reflect the sum of water loss via distinct skin components (interfollicular epidermis, HFs and sweat glands) and various skin-barrier related features (e.g., filaggrin expression, lipid composition or tight junctions). This lack of specificity may also prevent detailed insights into the molecular action of penetration enhancers or other barrier disrupting treatments.

#### 3.1.2. Transepithelial Electrical Resistance (TEER) and Electrical Impedance Spectroscopy (EIS)

TEER measures the resistance at direct current. It is commonly used in monolayer cell cultures grown on transwell filters [60,125] applying “chopstick” electrodes or EndOhm chambers. In recent years it was also used for 3D cultures of reconstructed human epidermis/skin [74,84,126,127] or human skin [128]. While this is a good tool to measure overall barrier function to ions, which means comprising transcellular and paracellular barrier as well as barrier of the SC and the viable epidermis including TJs, it is not able to distinguish between these barriers. To this end, more sophisticated EIS methods are needed.

EIS measures the resistance at alternating currents of different frequencies. Accessible are two-dimensional cell cultures and the measured impedance provide information on the paracellular passage of ions commonly referred to as barrier resistance, the transcellular capacity coupled current and the ventral distance between the cell and the cell binding substrate [129,130]. This technique enables the longitudinal recording of the epidermal barrier in real time. By using two-path impedance spectroscopy it is also possible to differentiate between paracellular and transcellular epithelial resistance [131]. Recently, EIS was used to measure the ion barrier of murine skin in vivo. Papain induced destruction of the skin barrier, as indicated by an impaired SC and disrupted TJs, resulted in a reduced resistance. Interestingly, the measured impedance correlated clearly with TEWL measurements [132]. However, the three-dimensional epidermis complicates the interpretation of the measured impedance and distinction between SC and TJ (and further barrier components) is not possible with the usual techniques. Additional mathematic modelling [133] and/or advanced techniques [134] are required to fully understand the obtained results. Drug or penetration enhancer-related changes of epidermal ion barrier are accessible by EIS. However, changes in EIS do not always correlate with the permeation rate of the drug/substance, as was e.g. shown by using different penetration enhancers to deliver theophylline, or determination of p-chloronitrobenzene flux after DMSO-induced skin damage [135,136].

#### 3.1.3. Chemical and Radiochemical Analyses

Several marker molecules to test skin barrier function as well as drugs can be detected and quantified by chemical analyses such as high performance liquid chromatography (HPLC), ultra-performance liquid chromatography (UPLC), liquid chromatography tandem-mass spectrometry (LC-MS/MS), ultra-high performance liquid chromatography tandem mass spectrometry (UHPLC-MS/MS) or high performance thin layer chromatography [137,138,139]. In addition, also radioactively labelled surrogates for hydrophilic (caffeine) or lipophilic (testosterone) molecules are often used [140]. To this end, the molecular markers/drugs are applied in vitro (reconstructed human epidermis/skin, e.g., [141,142]), ex vivo (e.g., excised skin in horizontal or vertical diffusion cells—e.g., Franz cells—, Saarbrücken penetration model, Hamburg model of penetration, [138,143,144]) or in vivo [145,146] onto the skin.

Often, molecular markers/drugs are detected after complete permeation of the skin by investigating blood levels or concentrations in the acceptor compartment of the diffusion (Franz) cell or in culture medium. Although measurements can be conducted at different time points, thus resulting in the ability to perform permeation kinetics, there is no information where drug diffusion was eventually decelerated within the skin.

To get more information about this item, the skin samples can be separated into different compartments by tape-stripping (allowing detailed analysis of the various SC layers), or sectioning (e.g., SC, epidermis and dermis) or heating (separation epidermis/dermis). The segregated compartments can be investigated independently [137,145,147,148]. This also allows the calculation of penetration kinetics, especially when using tape strips [149].

Even though these approaches to separate different skin compartments are already a big step forward, they still have several limitations: In vivo testing of drug delivery is not possible because a skin biopsy is needed for evaluation after drug application. In addition, these tests can only be performed at one point in time per tissue sample. Furthermore, barrier structures within a compartment (e.g., TJs within the epidermis) cannot be specifically addressed. Finally, it is difficult to distinguish between interfollicular epidermis and hair follicles and glands.

#### 3.1.4. Dye Permeation Analyses

Topical application of usually fluorescent dyes, such as Lucifer yellow and fluorescein isothocyanates (FITCs) of different sizes are used to measure kinetics of dye permeation in 2D cell cultures but also in reconstructed human epidermis and in skin [60,74,125,128,150] to determine skin barrier function. To this end, specimens are taken from the acceptor/basal compartment and measured using fluorescent readers. This method can also be used for fluorescent-(labelled) drugs or drug delivery systems, e.g., 5-amino-levulinic acid [121,151]. This is a very useful technique to quantify penetration over time. Yet, it does not discriminate between the various barriers of the skin. Combining this technique with microscopical techniques can visualize local concentrations of the dye [33,152] (see also Section 3.2).

#### 3.1.5. Raman Spectroscopy

Vibrational spectroscopy techniques such as Raman spectroscopy are common analytical tools in skin research [153,154]. They can also provide insights into molecular changes which are potentially related to skin barrier functions such as skin hydration [155]. Beside the examination of the skin physiology, Raman spectroscopy appears to be also suited for the tracking of drugs and to determine drug penetration depth and quantitative information on drug accumulation in the skin [156]. In principle, the collected data give information on chemical compounds within the skin and they can provide a means to track characteristic drugs without the need of labelling. Every substance could provide characteristic spectra, especially large delocalized electron systems produce comparable strong Raman signals. This has e.g., enabled the detection of ibuprofen, lidocaine or caffeine skin permeation [154,157,158]. However, the high complexity of skin generates also very complex Raman spectra blurring drug related signals and prevent a straightforward interpretation of the obtained results [156]. To overcome these limitations, the combination of Raman spectroscopy with high resolution microscopy techniques such as confocal laser scanning microscopy or multiphoton microscopy is in the focus of recent research [159]. The outstanding advantage of such Raman microscopy is the high spatial resolution in the µm range in all three dimensions and the non-invasive, label free profiling of the chemical skin composition [160,161]. What is more, the acquired intensity signals connected to the concentration of the probed molecules allowing quantitative analysis of the skin [162]. However, long acquisition times, high levels of background noise and time-consuming image analysis limit the widespread use of Raman microscopy in dermatological research. Coherent anti-Strokes Raman spectroscopy (CARS) is a modified technical approach that significantly increases the imaging speed. The development of the combined multiphoton/CARS microscope could be considered as an important step towards a clinical application of Raman spectroscopy [163]; yet, further quenching of background signals and automatized image analysis is required to grant a close implementation into routine examination in dermatology. Further progress in the development of user-friendly devices and automatized image acquisition and analysis will open a broader usage of Raman spectroscopy in (trans)dermal drug delivery research [164].

#### 3.1.6. Fourier Transform Infrared (FTIR) Spectroscopy

Another vibrational technique often used in combination with Raman spectroscopy is Fourier transform infrared (FTIR) spectroscopy. Compared to other spectroscopic methods, FTIR spectroscopy samples the absorbance of molecular vibrations resulting in characteristic fingerprints for many biomolecules. FTIR spectroscopy has numerous advances compared to classical dispersive spectroscopy [165,166]. First, the light yield is increased by up to factor 200 and therefore a much better signal-to-noise ratio can be achieved (the so called “Jacquinot” advantage). Second, the emission/absorption spectrum is not measured sequentially with regard to the wavelength but in parallel over the whole frequency range which, too, increases the signal-to-noise ratio (“Fellgett” or multiplex advantage). Third, the wavelength scale can be calibrated by a Helium-neon (HeNe) laser as a reference beam resulting in much better wavelength accuracy (“Connes” advantage). For real-time measurements of highly dynamic processes, fast scanning FTIR spectrometers with microsecond time-resolution are currently in development [167].

Attenuated total reflection (ATR) can be used as an extension to standard transmission FTIR spectroscopy enabling direct measurements of samples in a solid or liquid state without any preceding sample preparation and a penetration depth of 0.5 to 2 μm. For using ATR, the sample has to be brought in direct contact with special ATR crystals with a high refractive index (e.g., diamond).

Concerning the skin barrier, the molecular conformation of the lipid matrix within the SC is of special interest. Boncheva et al. provide a method for estimating the molecular lipid structure by using ATR-FTIR spectroscopy for the measurement of the CH2 scissoring bandwidth. Besides comparing the molecular organization of SC lipids in human, porcine and reconstructed skin, they estimate the changes following the topical application of penetration enhancers such as oleic acid [13]. In general, the method allows conclusions whether the intercellular lipids in the SC are mainly arranged in the orthorhombic, hexagonal or liquid lateral packing. In addition, the secondary structure of keratin can be described. Finally also water content in the skin can be delineated [168].

### 3.2. Microscopical Methods

**Figure 4:**
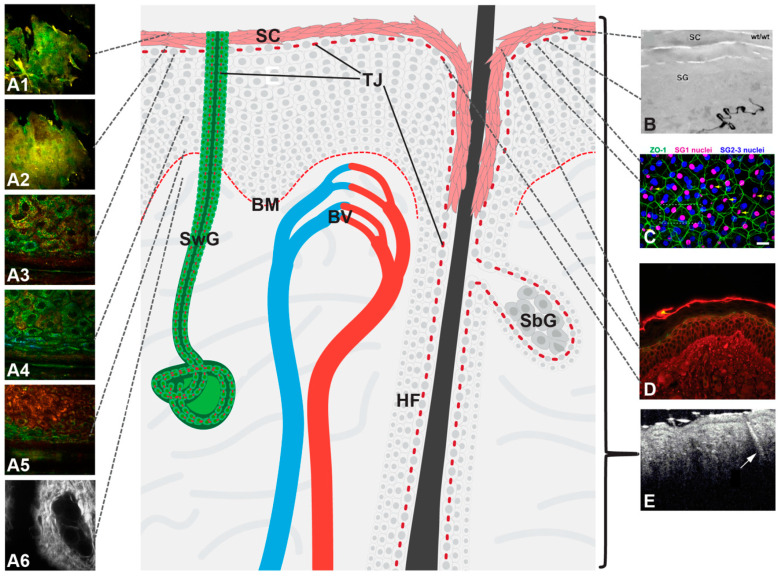
Localization of mechanical barriers in the skin (light red: stratum corneum (SC), Tight junctions (TJs) and basement membrane (BM)) and graphical presentation of the various microscopical methods described in this review: (**A1**–**A6**) Multiphoton microscopy with fluorescence lifetime imaging and second harmonic generation signal detection (**B**) transmission electron microscopy (**C**) confocal laser scanning microscopy of immunofluorescence stainings (**D**) Immunohistochemical analysis of a biotinylation assay (**E**) optical coherence tomography (OCT). The bracket denotes the area measured by OCT not discriminating between the different barriers within this area. Dotted black lines denote specific barriers/skin areas addressed by a method. Arrow in E denotes a hair follicle. BV: blood vessel, HF: hair follicle, SbG: sebaceous gland, SwG: sweat gland, (**B**) from [172], (**C**) from [170], (**E**) from [173].

The various methods described in this chapter are summarized in Table 1. Figure 4 gives an overview of the skin compartments addressed by the various microscopical methods and the barriers/skin areas that can be visualized.

#### 3.2.1. Immunohistochemical Analysis

To investigate TJ-related barrier function, the simultaneous analysis of molecular tracers which can be primarily or secondarily detected by fluorescence microscopy and that were injected into the dermis (or applied to the basal compartment of 3D cell cultures), in combination with labelling of distinct TJ proteins by antibody-linked immunofluorescence, have frequently been used in skin samples of different species, applying normal fluorescence microscopy or confocal laser scanning microscopy (CLSM) [64,65,74,97,109,169,170,171]. This method which is called “biotinylation assay” when using EZ-Link^TM^-Sulfo-NHS-LC-Biotin (Biotin-SH) gives insight into barrier function of TJs in interfollicular epidermis of healthy and diseased/knock-out skin [64,65,74,169,170], HFs [97,171] and glands [109]. This method warrants high molecular resolution of the localization of the stop of tracers of different sizes (up to now 556 Da (“Biotin-SH”), 1500 Da, 5000 Da, 31 kDa) in correlation with the TJ barrier. By using antibodies directed to SC components this could also be used to determine barriers within the SC from outside-in when tracers are applied topically or from inside-out in situations with impaired TJ barrier

However, even though this method is very valuable in basic research, it has several disadvantages to investigate drug delivery and thus plays, up to now, only a marginal role in drug formulation design. First of all, it can only be performed ex vivo or in vitro with processed tissue samples with processing for immunohistochemical stainings always including the possibility of changing the position of the drug investigated. In addition, it is not possible to perform successive measurements but only one point in time per sample can be investigated. Furthermore, quantification is, at least at the moment, time consuming [74]. Finally, the drug or the delivery system must be fluorescent or detectable by antibodies. Tagging the drug by a fluorescence dye might change the penetration behavior of the drug by changing its size and polarity and therefore the results may only give limited information on the penetration behavior of the drug itself. Thus, this technique might only be utilizable for very large drugs where the addition of the tag only results in minor changes. For 3D imaging, one could combine 3D tissue antibody staining [170] with CLSM/multiphoton microscopy (MPM), optimally with drugs that can be detected by fluorescence lifetime imaging (see Section 3.2.4 and Section 3.2.5).

#### 3.2.2. Transmission Electron Microscopy

Another approach to investigate detailed localization of the barrier function of the skin to tracers is to use electron dense tracers like lanthanum combined with ultrastructural demonstration of TJ and SC structures in electron microscopy [174]. However, this method includes even more processing and is more time consuming than the light microscopical approach mentioned above. Nonetheless, transmission electron microscopy was used for monitoring dermal penetration of e.g., nanoparticles with different outcomes [175,176].

#### 3.2.3. Optical Coherence Tomography (OCT) and Optoacoustic Imaging

OCT has been introduced in dermatology more than 20 years ago and proven as a useful tool for non-invasive morphological skin analysis [177,178]. It provides a large field of view (6 × 6 mm) and an optical penetration depth of up to 2 mm with a lateral and axial resolution in the 10 µm range [179]. Accordingly, OCT enables the discrimination between the SC and the living epidermis, papillary ridges and the subjacent dermis. Further advances resulted in the development of high definition (HD)-OCT. In contrast to OCT, HD-OCT provides an increased lateral and axial resolution in the 3 µm range whereas penetration depth and the field of view is decreased to approximately 1 mm and 2 mm, respectively. OCT and HD-OCT are valuable tools for diagnostic purposes and they are suitable to detect disease-related changes of the skin [179].

Currently, only few groups applied OCT to track the permeation of exogenously added agents through the skin. In this context, OCT was used to follow nanoparticle delivery through HFs [173,180] and epidermis [181]. OCT was also used to track glucose diffusion across the skin of rhesus monkeys [182]. Though, the majority of reports focused on micro needled-based dermal delivery systems in which OCT was used to visualize the position of the needles within the skin [183,184]. Further studies used OCT to document the effect of drugs on skin morphology which may only indirectly prove dermal drug delivery [185,186,187].

Optoacoustic or photoacoustic imaging is non-invasive and fast. It depends on the light illumination of the tissue trough e.g., pulsed lasers and the detection of light induced pressure waves through broadband ultrasound detectors. Upon multispectral light excitation, naturally occurring light adsorbers such as melanin or hemoglobin generate characteristic spectra which enable their discrimination in the tissue [188]. Real-time imaging with handheld devices envisions the broad clinical application of optoacoustic instruments in the near future [189]. Optoacoustic imaging provides a three-dimensional view into the skin with an imaging depth ranging from several centimeters to micrometers and a spatial resolution ranging from hundreds of micrometers to hundreds of nanometers, respectively. This is in contrast to conventional light microscopic approaches, where immense light scattering prevent access to deeper tissues. Therefore, optoacoustic imaging allows to differentiate between single skin layers (SC, viable epidermis and dermis) and enables visualization of the blood capillary loops and the vascular plexus (impact of the vascular barrier on drug delivery). Addressing cancer-induced morphologic (e.g., angiogenesis) or metabolic changes (e.g., tissue glycation) within the skin, several studies already document the applicability of optoacoustic imaging for diagnostic purposes [190,191,192] or potentially cancer-related changes of tissue glycation [193].

Although not required for imaging, light adsorbing substances such as fluorophores can be identified by optoacoustics enabling the in vivo tracking of drug permeation through the epidermis towards the blood circulation. Parallel assessment of disease related features such as epidermis thickness or vasodilatations [159] offer a potential tool that combines morphological skin layer evaluation, disease severity and drug permeation. To our knowledge there is no current study investigating (trans)-dermal drug delivery by optoacoustic imaging. However, especially from cancer-related research, several examples showed the use of optoacoustic imaging for the detection of fluorescently labelled drugs or drug carriers, like Cy7 conjugated iron oxide nanoparticles (Cy7-SPION) or PEGylated indocyanine green (ICG) liposomes, within experimental tumors such as breast cancer [194,195,196]. Interestingly, recent developments suggest the applicability of optoacoustic induced power waves to transiently open the SC and thus to facilitate drug permeation. Further developments may provide devices that allow diagnostic imaging, promote drug penetration and simultaneous tracking of the drugs [197].

#### 3.2.4. Confocal Laser Scanning Microscopy (CLSM)

CLSM and especially confocal fluorescence microscopy can be a valuable tool to investigate the skin barrier and the delivery of drugs in vitro, ex vivo and in vivo. It can be very beneficial for the investigation of immunofluorescence stainings in 3D samples [170] (see also Section 3.2.1). Compared to classical bright field and fluorescence microscopy, CLSM uses a focused laser beam to excite the sample point-wise on a raster in *x* and *y* direction. The emission is then spatially filtered by a confocal pinhole to block out-of-focus light, sacrificing image intensity in exchange for improved image contrast compared to bright field microscopy. Image acquisition at multiple steps in *z* direction and subsequent three-dimensional reconstruction allows for investigation of skin structures in all three space dimensions.

Besides imaging of endogenous fluorophores like reduced nicotinamide adenine dinucleotide (NADH), collagen or melanin, to illuminate the spatial structure of the skin, dermal drug delivery can be probed by fluorescence labelling of the drug or—in special cases, if the drug itself has fluorescent properties—without labelling [198].

For example, Alvarez-Román et al. investigated the skin penetration and distribution of polymeric nanoparticles in porcine skin ex vivo by CLSM and observed an accumulation in HFs and skin furrows [199].

Another application of CLSM is reflectance confocal microscopy (RCM), which uses backscattered light instead of fluorescence emission for imaging. In vivo RCM is frequently used as a diagnostic tool in dermatological routine [200], but has also many potential use cases in skin barrier research due to almost real-time imaging capabilities and relatively modest acquisition costs.

#### 3.2.5. Multiphoton Microscopy (MPM)

After its first operability demonstration 30 years ago in a laboratory setting [201], MPM overcomes the limit of explorative practice and developed as a powerful instrument in the area of skin basic research and increasingly clinical applications [202,203].

Three photonic component parts define MPM’s applicability for the characterization of human skin.

Based on the nonlinear excitation of naturally occurring endogenous fluorophores such as NADH, flavin adenine dinucleotide, tryptophan, porphyrins and melanin [204], and protein structures (e.g., elastin, collagen) [205,206], the morphological pattern and layering of the skin can be evaluated on a subcellular level. Beyond the reflection in the outermost barrier of the epidermis—the SC—the first living cell in the viable epidermis can be assessed in detail with the incidental finding of a precise discrimination of the SC/SG transition. The principle of two-photon excitation overcomes the limitations of fluorescence imaging, e.g., operation of CLSM in fluorescence mode, for innocuous in vivo utilization. Here, with a negligible linear energy transfer at the boundary layer, the excitation almost exclusively takes place within the target volume of examination without damaging the surrounding tissue [207].

The additional utilization of the nonlinear optical process second harmonic generation (SHG), observed in noncentrosymmetric molecular structures such as collagen [163,208], allows, next to its application in the area of dermal fibril characterization up to a penetration depth of approximately 200 µm [209,210,211], the exact discrimination of the dermo–epidermal junction and consecutively the epidermal thickness.

Third, MPM additionally offers the applicability of fluorescence lifetime imaging microscopy (FLIM) [163,212]. Since the lifetime of a fluorophore—the time it spends in the excited state before returning to the ground state by photon emission—is characteristic for a fluorophore in its biochemical environment [213,214], FLIM facilitates a distinct in vivo identification of fluorescent molecules that share an overlap in their morphological or spectroscopic pattern within human skin. By time-correlated single photon counting [213] or phasor analysis [215] selective visualization of endogenous fluorophores or topical drug uptake was successful at high spatial resolutions [216].

Therefore, MPM FLIM enables for non-invasive high-resolution examination of human skin in vitro, ex vivo and in clinical in vivo applications. On this basis, clinical studies focused on both high-resolution morphological patterns and pathophysiological variances on a cellular level e.g., in the process of skin wound healing [217,218]. Next to MPM FLIM application in advanced skin cancer diagnosis [203,219], also the skin barrier function itself became the focus of attention: Volz as well as Frombach used high-resolution Cluster-FLIM in in vitro- and ex vivo-approaches to explicitly inspect the dynamics of both drug penetration and the penetrated skin area itself [163,216,220,221,222].

Transferring skin barrier analysis to ‘patient’s bedside’, Ogawa-Fuse examined skin hydration utilizing a combination of already mentioned techniques such as CARS/Raman and MPM FLIM [220]. Moreover, the same technical combination facilitated the measurement of pharmaceutical and cosmetic drug penetration and delivery through the skin, e.g., minocycline and tazarotene [163,216,221,222].

The combination of mentioned non-invasive spectroscopic and microscopic techniques such as CARS/Raman spectroscopy, MPM and associated techniques provide a huge potential for the investigation of the skin barrier and the related impact on dermal or even transdermal drug delivery. Sophisticated image analysis through artificial intelligence may open up the broad application of high-resolution skin microscopy in skin research and clinics [164].

## 4. Outlook/Conclusions

The present review highlights current knowledge on skin barriers that have to be overcome by a drug or its carrier system and tools that allow the localization and quantification of skin barrier functions, skin barrier components or tracking of tracer molecules on their way through the skin from outside to inside and from inside-out. The SC is considered as the most efficient barrier that controls the entrance and permeation of topically applied substances. TJs are a second barrier, especially important when SC is impaired—either by e.g., skin diseases or due to penetration enhancers—or for drugs not limited by the SC. However, also other parts of the skin may limit or decelerate drug diffusion and systemic delivery. For example, the basement membrane at the interface between dermis and epidermis as dense mesh of structural proteins and carbohydrates or the blood vessels underneath the membrane may play a role in the overall barrier system of the skin. To investigate the skin barrier and to discriminate between different barrier-forming components various tools are available. Immanent limitations of every method have to be considered for proper interpretation of obtained research data. Recent advances in high resolution imaging (e.g., Raman microscopy, optoacoustic imaging and MPM-FLIM) provides pioneering technologies for an improved understanding of the skin barrier on the one hand and dermal drug delivery on the other hand. Broad application of those novel techniques is currently limited because of sophisticated image analysis required for accurate data interpretations. One solution for most of these hurdles can be the application of advanced machine learning techniques. Especially automatic image segmentation and structure recognition based on supervised learning of deep neural networks show promising results on many tasks if the required amount of annotated training data is available or can be generated without too much effort. Steady progress in skin barrier research and increasing interest in (trans) dermal drug delivery will push forward the development of even more delicate instruments that extend the toolbox of dermatological and pharmaceutical research. Among many other interesting methods, advances in electrical impedance spectroscopy, Cluster-FLIM, X-ray or stimulated Raman spectromicroscopy [134,223,224] envision an improved view on skin barrier functions and associated structures as well as the simultaneous tracking of distinct molecules within the skin.

## Figures and Tables

**Table 1:** Summary of analytical tools to investigate skin barrier and their potential use to measure flux.

Method	Can Distinguish between Interfollicular Epidermis, Hair-Follicles and Glands	Can Distinguish between Barriers in SC, Viable Epidermis, Dermis	Can Specifically Address Tjs in the Viable Epidermis/Hair Follicles/Glands	Measurement of Inside-Out or Outside-In Flux?	Flux Can Be Quantified?	Measurement at Different Time Points Possible [Permeation-Penetration Kinetics]?	Human In Vivo ^1^ Application Possible?
TEWL	−	−	−	Inside-out	+	++	++
TEER	−	−	−	No directionality	++	++	−
Advanced electrical impedance spectroscopy	−	+	−	No directionality	++	+	(+)
(Radio)Chemical analyses of drugs/tracers applied onto the skin in acceptor compartment/blood (e.g., by UPLC, UHPLC-MS/MS etc.)	−	−	−	Outside-in	+++	++	+
Chemical analysis of drugs/tracers applied onto the skin in different skin layers after separation ^2^ and subsequent extraction (analyses e.g., by UPLC, UHPLC-MS/MS etc.)	−	(+) (with certain limitations)	−	Outside-in	+++	− [++] ^3^	−
Dye permeation analysis	−	−	−	Outside-in	++	++	−
Raman Spectroscopy/microscopy	+	+ ^4^/++ ^5^	−	Outside-in and inside-out ^6^	+	++	++
(ATR-)FTIR spectroscopy	(+) ^7^	(+) ^7^	−	Outside-in and inside-out ^6^	+	++	++
Transmission electron microscopy combined with tracers	+++	+++	+++	Outside-in and inside-out ^6^	−	−	−
Immuno-histochemistry combined with tracers (e.g., biotinylation assay) (Fluorescence microscopy or CLSM)	++	++	++	Outside-in and inside-out ^6^	(+)	−	−
OCT/optoacoustic imaging	+	+	−	Outside-in and inside-out ^6^	+	++	++
In vivo CLSM/reflectance confocal microscopy (RCM)	+ (HF) − (Glands)	−	−	Outside-in and inside-out ^6^	−	++	+
MPM/FLIM	+	++	(−) except for intrinsic fluorescent TJs	Outside-in and inside-out ^6^	(+)	++	+

^1^ in vivo means measurement in vivo is possible without taking biopsies, ^2^ e.g., by tape stripping, horizontal sectioning or heat treatment; ^3^ determination of penetration kinetics possible by e.g., evaluation of consecutive tape strips and constructing curves of absorption, ^4^ Raman spectroscopy, ^5^ Raman microscopy, ^6^ depending on the site of application, ^7^ normally used for SC only.
